# Hyperbaric oxygen protects against periodontal bone loss by modulating inflammation and bone remodeling via RANKL/OPG expression in ligature-induced periodontitis

**DOI:** 10.7150/ijms.122857

**Published:** 2026-01-01

**Authors:** Kang-Wei Tu, Chien-Cheng Huang, Mao-Tsun Lin, Ko-Chi Niu, Cheng-Hsien Lin, Pi-Yu Chao, Ching-Ping Chang, Jimmy Lian Ping Mau

**Affiliations:** 1 Department of Periodontics, Chi Mei Medical Center, Yongkang, Tainan 710, Taiwan; Department of Environmental and Occupational Health, College of Medicine, National Cheng Kung University, Tainan 70428, Taiwan.; 2 Department of Emergency Medicine, Chi Mei Medical Center, Tainan 710, Taiwan; School of Medicine, College of Medicine, National Sun Yat-sen University, Kaohsiung, Taiwan; Department of Emergency Medicine, Kaohsiung Medical University, Kaohsiung, Taiwan.; 3 Department of Medical Research, Chi Mei Medical Center, Tainan 710, Taiwan.; 4 Department of Hyperbaric Oxygen, Chi Mei Medical Center, Tainan 710, Taiwan.; 5 Department of Medicine, Mackay Medical University, New Taipei City, Taiwan.; 6 Department of Periodontics, National Cheng Kung University Hospital, Tainan 704302, Taiwan; Department of Periodontics, Tri-Service General Hospital, Taipei 114202, Taiwan; Yuya Dental Clinic, Tainan 709020, Taiwan; Department of Senior Services, Southern Taiwan University of Science and Technology, Tainan 710301, Taiwan.

**Keywords:** periodontitis, hyperbaric oxygenation, RANKL, osteoprotegerin, inflammation, bone resorption

## Abstract

Periodontitis (PD) is a chronic inflammatory disease characterized by the accumulation of bacterial metabolites, sustained immune activation, and progressive loss of alveolar bone. Hyperbaric oxygen therapy (HBOT) has demonstrated anti-inflammatory and bone-reparative properties; however, its mechanistic effects in periodontitis remain underexplored. This study investigated whether HBOT mitigates periodontal bone loss and modulates bacterial, inflammatory, and osteoclastogenic pathways in a ligature-induced rat model. Sixty male Wistar rats underwent ligature placement for 28 days and were allocated into five groups (Sham, PD, PD+ natural recovery [RECOV], PD+early HBOT [EHBOT], PD+late HBOT [LHBOT]); HBOT was delivered as 100% oxygen at 2.0 ATA for 60 min/day. Gingival tissues were assessed for bacterial metabolites, lipoteichoic acid (LTA) and lipopolysaccharide (LPS), inflammatory cell infiltration, fibrotic integrity, and alveolar bone resorption. Cytokine and chemokine arrays were performed to evaluate cytokine-induced neutrophil chemoattractants, interleukin-1α, interleukin-1β, interleukin-1 receptor antagonist, LPS-induced chemokine CXCL5, thymus chemokine, tissue inhibitor of metalloproteinases-1, soluble intercellular adhesion molecule-1, and L-selectin. Ligature-induced periodontitis triggered robust inflammatory responses, elevated bacterial burden, increased receptor activator of nuclear factor kappa-B ligand (RANKL), and suppressed osteoprotegerin (OPG), promoting osteoclastogenesis and bone loss. Importantly, EHBOT produced more pronounced reductions in LTA/LPS and pro-inflammatory mediators and yielded greater preservation of trabecular microarchitecture than LHBOT or RECOV. HBOT overall significantly reduced LTA/LPS levels, suppressed inflammatory cytokines and adhesion molecules, and restored the RANKL/OPG balance in osteoblasts and osteocytes. Histological and micro-computed tomography analyses confirmed that HBOT preserved trabecular bone microarchitecture. These findings highlight the multi-targeted therapeutic potential of HBOT in suppressing inflammation, limiting immune cell infiltration, and preventing bone destruction, supporting its use as an adjunctive intervention for periodontitis and inflammatory bone disorders.

## Introduction

Periodontitis is a chronic inflammatory disease characterised by the progressive destruction of gingival tissues and alveolar bone, primarily triggered by the accumulation of dental plaque and pathogenic bacteria 1, 2. The resulting host immune response leads to the production of pro-inflammatory cytokines and proteolytic enzymes, which contribute to tissue breakdown and the activation of osteoclasts, responsible for bone resorption 3. Among bacterial components, lipoteichoic acid (LTA) and lipopolysaccharide (LPS) act as ligands for toll-like receptors 4, exacerbating local inflammation and promoting osteoclastogenesis 5.

A key mechanism in bone resorption involves the receptor activator of nuclear factor kappa-B ligand (RANKL), which binds to its receptor RANK on osteoclast precursors to stimulate their differentiation and activity 6. This process is counteracted by osteoprotegerin (OPG), a decoy receptor that neutralises RANKL 7. The balance between RANKL and OPG is thus a critical determinant of osteoclast activity and periodontal bone homeostasis.

Conventional therapy, scaling and root planing (SRP) with or without adjunctive antimicrobials, reduces microbial burden but can be limited in hypoxic, poorly perfused pockets, in sites with high inflammatory load, and in patients with systemic risk factors; routine antibiotic use also raises concerns about resistance and dysbiosis 8-11. These constraints motivate development of oxygen- and perfusion-targeted adjuncts that can complement mechanical debridement by improving the tissue milieu for resolution and repair.

Hyperbaric oxygen therapy (HBOT), intermittent inhalation of 100% oxygen at elevated ambient pressure and increases tissue pO_2_, reduces edema via hyperoxic vasoconstriction, and modulates inflammatory and redox signalling 12-14. Reports in oral and craniofacial contexts (e.g., compromised flaps, osteomyelitis, osteonecrosis) and in bone-healing models suggest that HBOT can lower cytokine expression, improve microcirculation, and favour osteoblast activity 15-18. However, prior studies typically assessed narrow endpoints and rarely linked microbial ligands (LTA/LPS), leukocyte trafficking/adhesion, and bone-remodelling signals (RANKL/OPG) to structural outcomes within a single framework, nor did they test whether treatment timing alters efficacy.

Here, using a ligature-induced periodontitis model in rats 19, we evaluated whether HBOT mitigates periodontal inflammation and bone loss by integrating multiple levels of readouts: bacterial metabolites (LTA/LPS), cytokine/chemokine and adhesion programmes, cellular infiltration, RANKL/OPG expression in osteoblasts/osteocytes, and micro-CT-defined trabecular architecture. Crucially, we compared early versus late HBOT initiation against natural recovery to test schedule sensitivity of the response. We hypothesised that HBOT would reduce LTA/LPS burden and inflammatory signalling, restore the RANKL/OPG balance, and preserve alveolar bone microarchitecture, thereby providing a mechanism-linked rationale for HBOT as an adjunct to standard periodontal therapy.

## Materials and Methods

All experimental procedures were approved by the Institutional Animal Care and Use Committee (IACUC) of Chi Mei Medical Center, Tainan, Taiwan (Approval No. 110122317), and were conducted in accordance with the Guide for the Care and Use of Laboratory Animals (8th edition, National Academies Press, 2011). The study adhered to the ARRIVE (Animal Research: Reporting of *In Vivo* Experiments) guidelines to ensure ethical and transparent animal research practices.

Sixty 7-week-old male Wistar rats (250-350 g) were obtained from BioLASCO Co., Ltd. (Taipei, Taiwan) and housed in groups of four per cage under controlled environmental conditions (temperature 26 ± 0.5°C, 12-hour light-dark cycle). Rats were provided with food and water ad libitum and acclimated for at least two weeks before experimental procedures commenced.

A detailed protocol, including the research question, experimental design, and analysis plan, was developed prior to the study to ensure consistency and methodological rigor, although it was not formally registered in a public repository. All efforts were made to minimize animal suffering and reduce the number of animals used while ensuring scientific validity.

### Periodontitis (PD) rat model

Adult male Wistar rats were anesthetized with an intraperitoneal dose of Zoletil®100 (20 mg/kg; Virbac, Carros, France) and Rompun® (xylazine, 10 mg/kg, Bayer, Leverkusen, Germany). As described in a previous study 19, 20, sterile 4-0 black braided silk threads (Surgical silk sutures, UNIK 25YD (23M10034, Taipei, Taiwan) were tied around the cervix of the maxillary second molar and mandibular first molar bilaterally to induce experimental periodontitis. Under a stereomicroscope, we ensure all the ligature placements are lodged at the proper subgingival position. Rats in which the ligature could not be successfully positioned around the molars or became dislodged prematurely, thereby compromising the induction of periodontitis, were excluded from the study. However, none of the animals met the exclusion criteria, as ligature placement and retention were successfully achieved in all cases.

### HBOT procedure

**Figure 1:**
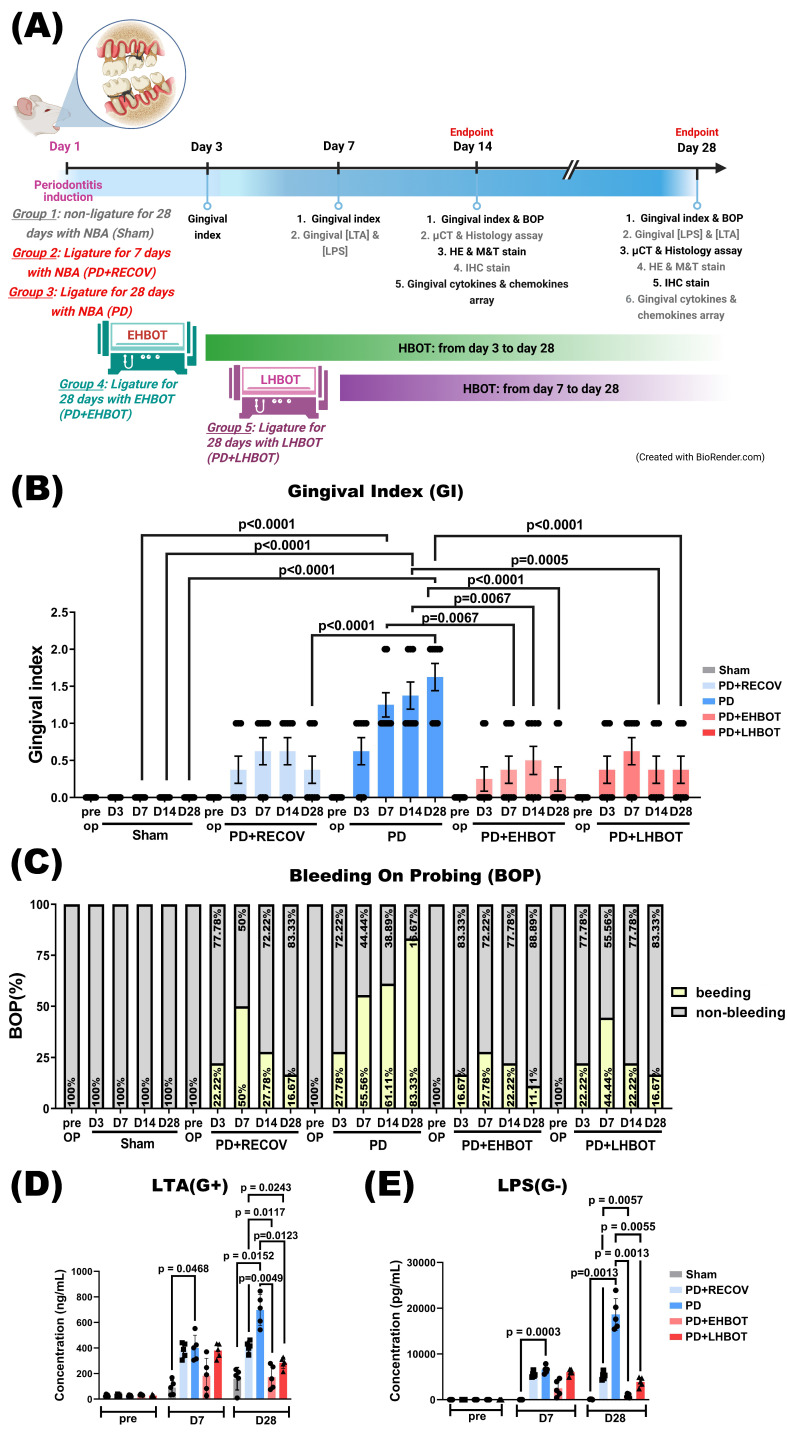
** Effects of HBOT on ligature-induced periodontitis. (A)** Experimental groups and procedures. Rats were assigned to five groups: (1) Sham: unligated rats without HBOT exposure; (2) PD+RECOV: rats subjected to ligature placement for 7 days without HBOT; (3) PD: rats subjected to ligature placement for 28 days without HBOT; (4) PD+EHBOT: rats subjected to ligature placement for 28 days with HBOT administered from day 3 to day 28; and (5) PD+LHBOT: rats subjected to ligature placement for 28 days with HBOT administered from day 7 to day 28. The gingival index (GI) and bleeding on probing (BOP) were assessed on days 7, 14, and 28 post-surgery. Gingival concentrations of lipoteichoic acid (LTA) and lipopolysaccharides (LPS) were measured on days 7 and 28. Histological staining and micro-CT imaging were performed on days 14 and 28. Effect of HBOT on **(B)** GI, **(C)** BOP, and periodontal concentrations of **(D)** LTA and **(E)** LPS in the maxillary second molar and mandibular first molar across study groups. Abbreviations: PD, periodontitis; RECOV, recovery; EHBOT, early hyperbaric oxygen therapy; LHBOT, late hyperbaric oxygen therapy.

Animals assigned to HBOT received 100% oxygen at 2.0 atmospheres absolute (ATA) for 60 minutes once daily, delivered in a customized small-animal chamber (Chun Hung International Biomedical Technology, Tainan, Taiwan) as previously described 21. We selected 2.0 ATA for 60 min/day because (i) this pressure-time product is widely adopted in preclinical and clinical protocols with favorable safety margins 22; (ii) our prior laboratory experience with ischemia/hypoxia and reperfusion models showed reliable anti-inflammatory and pro-remodeling effects under this regimen 23, 24; and (iii) pilot feasibility work indicated clearer effect sizes than lower pressure exposures while avoiding signs of oxygen toxicity in rats under our monitoring protocol. Compression and decompression were performed gradually per the manufacturer recommendations. Non-HBOT animals were placed in the chamber under normobaric air (NBA; 21% O₂ at 1.0 ATA) for an equivalent duration. Animals were monitored during sessions; exclusion criteria included persistent respiratory distress, signs of ear barotrauma, neurologic abnormalities, or clinical features suggestive of oxygen toxicity; excluded animals were replaced to maintain prespecified group sizes. HBOT timing definitions: “Early HBOT” denotes initiation during the initial inflammatory phase (Day 3 after ligature; D3-D28, 26 sessions), whereas “Late HBOT” denotes initiation during the established disease phase (Day 7 after ligature; D7-D28, 22 sessions). For clarity, the timing windows, session numbers, and group allocation are summarized in **Table 1** and schematized in **Figure 1A**. Animals not assigned to HBOT received normobaric air exposures as handling controls.

### Experimental groups and procedures

Rats were randomized (computer-generated sequence via https://www.randomizer.org/) into five groups: (1) Sham: unligated, NBA exposure; (2) PD+RECOV: ligature for 7 days followed by removal and observation without HBOT; (3) PD: ligature for 28 days without HBOT; (4) PD+EHBOT: ligature for 28 days with HBOT D3-D28; and (5) PD+LHBOT: ligature for 28 days with HBOT D7-D28. Each group was evaluated at Day 14 and Day 28 (see **Table 1** for session counts). The primary outcome for power estimation was alveolar bone loss quantified as CEJ-ABC distance on micro-CT. Sample size was determined a priori using G*Power (α = 0.05, power = 0.80), yielding a total of N = 60 (n = 12 per group; 6 per time point), in accordance with ARRIVE. Allocation concealment and blinding were maintained: assignments were made by an investigator not involved in data collection or analysis; procedural personnel knew assignment solely to deliver interventions; all outcome assessments (histology, micro-CT, biochemical assays) and statistical analyses were conducted blinded to group.

### Gingival index (GI) and bleeding on probing (BOP)

Gingival health was evaluated using the Gingival Index (GI) and bleeding on probing (BOP) criteria 25, 26. Gingival bleeding was assessed within 30 seconds after probing with a Williams periodontal probe (PW, Hu Friedy) at all teeth to evaluate the GI and BOP. The GI was scored based on four degrees of severity: (1) score 0: normal gingiva, (2) score 1: slight inflammation with mild redness and edema, (3) score 2: moderate inflammation with redness and edema, and (4) score 3: severe inflammation with pronounced redness, edema, and spontaneous bleeding. Furthermore, the BOP was recorded at six sites per tooth and calculated as the percentage of bleeding sites. These scoring systems are widely validated for assessing gingival inflammation.

### Assessment of bacterial metabolites

The periodontal tissues, including the gingiva, periodontal ligament, and a portion of the alveolar bone, were excised, homogenized on ice in lysis buffer, and centrifuged to obtain clarified supernatants. Gram-positive lipoteichoic acid (LTA) and Gram-negative lipopolysaccharide (LPS) concentrations were quantified using commercially available immunoassays according to the manufacturers' instructions. Samples and standards were run in duplicate; curves were fitted with a four-parameter logistic model. Analyte concentrations were normalized to total protein (e.g., ng · mg⁻¹ protein) measured from the same supernatants. Blank controls were included for quality control.

We selected LTA as the Gram-positive proxy because it is membrane-anchored yet readily shed, allowing reliable recovery from tissue homogenates, whereas wall teichoic acid (WTA) is peptidoglycan-bound and requires harsh extraction 27; LPS was used as the Gram-negative proxy because it is the major outer-membrane glycolipid, shed freely and via outer-membrane vesicles 28.

Values were interpreted as community-level microbe-associated molecular patterns (MAMP) burden rather than species-specific measures. Representative oral taxa include LTA (G⁺): *Streptococcus mutans, S. sanguinis, S. gordonii, S. oralis; Actinomyces naeslundii/viscosus; Lactobacillus spp.; Enterococcus faecalis; occasionally Staphylococcus aureus* 29-31; LPS/LOS (G⁻): *Porphyromonas gingivalis, Tannerella forsythia, Prevotella intermedia/nigrescens, Fusobacterium nucleatum, Aggregatibacter actinomycetemcomitans, Campylobacter rectus, Capnocytophaga spp., Eikenella corrodens; spirochete Treponema denticola* produces lipooligosaccharide (LOS) with strong TLR2 activity 32-35.

### Cytokine and chemokine antibody array analysis

Gingival tissues surrounding the ligated and contralateral molars were harvested at days 14 and 28, homogenised in lysis buffer (RayBiotech, USA) supplemented with protease inhibitors, and centrifuged to collect supernatants. Total protein concentrations were quantified using a bicinchoninic acid (BCA) assay (Thermo Fisher Scientific, USA).

Protein extracts (200 µg per sample) were applied to Rat Cytokine Antibody Arrays (ARY008, R&D Systems, Inc., USA), which detect a panel of 34 inflammatory cytokines, chemokines, and adhesion molecules. For this study, data interpretation focused on the following targets relevant to periodontitis pathophysiology: CINC-1 (cytokine-induced neutrophil chemoattractant-1 / also known as CXCL1), CINC-2α/β (cytokine-induced neutrophil chemoattractant-2 alpha/beta / also known as CXCL3), sICAM-1 (soluble intercellular adhesion molecule-1), IL-1α (interleukin-1 alpha), IL-1β (interleukin-1 beta), IL-1ra (interleukin-1 receptor antagonist), LIX (lipopolysaccharide-induced CXC chemokine / CXCL5), L-selectin (leukocyte adhesion molecule CD62L), Thymus chemokine (also known as CCL25 or thymus-expressed chemokine), and TIMP-1 (tissue inhibitor of metalloproteinases-1).

The arrays were processed according to the manufacturer's protocol. Briefly, membranes were blocked, incubated with samples overnight at 4 °C, washed, and incubated with biotin-conjugated antibodies followed by horseradish peroxidase-conjugated streptavidin. Signals were visualised using enhanced chemiluminescence (ECL) and quantified using ImageJ software (NIH, USA). Densitometric analysis was performed for each cytokine/chemokine spot, and mean pixel intensity was normalised to the internal positive control. Data were expressed as relative expression compared to the sham control group. Membranes were imaged as single, intact exposures. Main-text panels were border-cropped only to remove non-informative margins; no repositioning or compositing was performed. Full, uncropped images and original films are provided in **Supplementary Figure S1**.

### Micro-computerized tomography (Micro-CT or μCT) imaging

A micro-CT Skyscan 1276 CMOS edition system (Bruker, Kontich, Belgium) assessed the morphology around the ligated maxilla second molar and ligated mandible first molar in all dimensions. The distances between the cementoenamel junction (CEJ) and the alveolar bone crest (ABC) were defined as the line passing through the distal cusp and perpendicular to the CEJ 36, 37. Both buccal and palatal sides of the upper maxilla second molar and lower first molars were measured in millimeters bilaterally using a CT Analyzer Skyscan software (Bruker, Germany). The alveolar bone loss was evaluated by measuring the average (CEJ-ABC) distance. Parameters including bone volume/total volume (BV/TV), bone surface/ bone volume (BS/BV), trabecular thickness (Tb.Th), trabecular separation (Tb.SP), and trabecular number (Tb.N) were determined.

**Figure 2:**
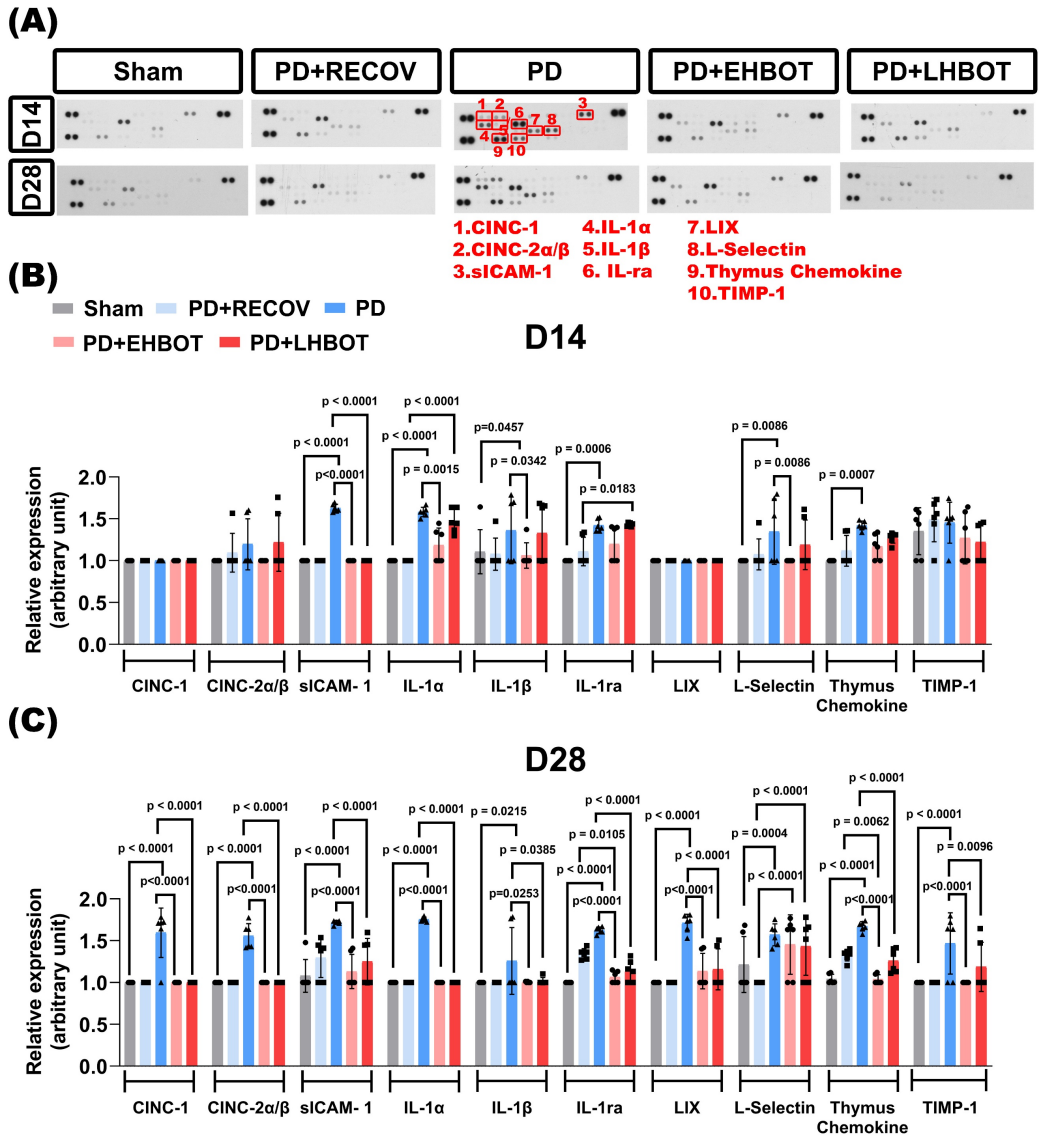
** HBOT reduces gingival cytokine and chemokine expression in ligature-induced periodontitis. (A)** Representative cytokine array blots showing expression profiles of 10 key inflammatory mediators (red boxes) in gingival tissues collected at day 14 and day 28 from Sham, PD+RECOV (natural recovery), PD (periodontitis only), PD+EHBOT (early HBOT), and PD+LHBOT (late HBOT) groups. The targeted proteins included: 1. CINC-1 (CXCL1), 2. CINC-2α/β (CXCL3), 3. sICAM-1, 4. IL-1α, 5. IL-1β, 6. IL-1 receptor antagonist (IL-1ra), 7. LIX (CXCL5), 8. L-selectin (CD62L), 9. Thymus chemokine (CCL25), and 10. TIMP-1. **(B)** Quantitative analysis of cytokine and chemokine expression at day 14. Periodontitis induced marked increases in several pro-inflammatory cytokines and chemokines. Early HBOT significantly suppressed most inflammatory mediators compared to the PD and PD+RECOV groups.** (C)** Quantitative analysis at day 28. PD-induced cytokine elevation persisted, while both early and late HBOT treatments effectively reduced IL-1α, IL-1β, IL-1ra, sICAM-1, CINC-1, CINC-2α/β, LIX, and thymus chemokine levels. Notably, early HBOT more effectively reduced thymus chemokine expression than natural recovery. Data are presented as mean ± SD. n = 6 per group. Panel 2A shows a cytokine dot-array membrane (R&D Systems ARY008) from a single, intact exposure; the published panel was border-cropped only to remove blank margins (global linear contrast, no compositing). See **Supplementary Figure S1** for the full, uncropped membrane and original X-film/RAW.

Following micro-CT analysis, both the maxilla and mandible specimens were carefully dissected to maintain structural integrity. To eliminate residual soft tissues, the samples were immersed in a sodium hypochlorite solution for 4 hours, followed by thorough mechanical cleaning. The specimens were then stained with methylene blue dye (1 g/100 mL; Sigma, MO, United States) for 5 minutes to facilitate the quantification of alveolar bone loss. Alveolar bone loss was assessed by measuring the linear distance from the CEJ to the ABC along the molar roots. Stained specimens were examined using a stereomicroscope (Zeiss) at 25x magnification. Measurements were taken on the maxillary second molar and mandibular first molar, repeated three times per specimen, and the mean values were used for statistical analysis. All evaluations were performed by an investigator blinded to the group allocations to minimize bias. Representative methylene blue-stained images are shown in **Fig. 2A**, depicting the buccal and palatal aspects of the maxilla.

### Assessment of inflammatory cell infiltration (ICI) and fibrosis

Samples were dehydrated, embedded in paraffin, sectioned along the molars in a mesiodistal plane, and stained with hematoxylin-eosin (H&E) and Masson's trichrome.^9^^38^ Semiquantitative scoring as: no visible ICI = 0, slightly visible ICI = 1, and dense visible ICI = 2, was performed to determine ICI. The scoring system was as follows: no visible fibrosis = 0, slightly visible fibrosis = 1, and dense visible fibrosis = 2, used to determine the degree of fibrosis.

### Bone histology observation

After micro-CT analysis, both the maxilla and mandible were obtained. The specimens (including gingivae, teeth, and bones) around the molars were dissected, fixed in 10% buffered neutral formalin for 24 h, and decalcified in a decalcifier system (DecalciferⅡ3800460, Leica, USA) for 16h. Specimens were stained with methylene blue (MB) dye (1 g/100 mL) for 1 min to quantify alveolar bone loss by measuring the linear distance of the cementoenamel junction (CEJ) from the alveolar bone crest (ABC) in molar roots.

Each sample was embedded in paraffin wax and sliced into 4μm thick sections in mesiodistal directions. Sections were mounted on glass slides and stained with Hematoxylin and eosin (H&E), Masson's trichrome, and tartrate-resistant acid phosphatase (TRAP) for microscopic examination.

The H&E-stained slices were measured using an optical microscope (Zeiss Axio Imager A2, Zeiss, Gmbh, Gottingen, Germany) with an AxioVision Imaging System (Zeiss) to observe the obstruction of the alveolar bone and periodontal tissue in crown and root sections and to monitor possible histological changes from normal tissues.

Masson's trichrome staining (Sigma-Aldrich, MA, USA) was performed to distinguish the bone matrix and defects. It has a strong affinity to essential proteins, notably collagen fibrils. The bluish color indicates collagen fibrils, mainly Type I collagen, in the bone matrix, whereas the bone defects show hollow images from being filled with hydroxyapatite.

TRAP immunohistochemical staining (Sigma-Aldrich) was performed on these sections with an anti-TRAP antibody and an immunostaining kit to identify osteoclast-like cells covering the bone surface. The osteoclasts were identified by TRAP staining near the bony resorptive lacunae and multiple nuclei (≥three).

### Bone immunofluorescence staining

Sections were treated with 3% hydrogen peroxide and then incubated with primary antibodies such as sclerotin (the osteocyte marker; 1:300, Abcan, Cambridge, UK), osterix (the osteoblast marker, 1:300, Abcam, Cambridge, UK), anti-osteoclast-associated receptor (OSCAR, the osteoclast marker; 1:300, Abcam, Cambridge, UK), anti-matrix-metallopeptidase-9 (MMP-9, the osteoclast marker; 1:300, Abcam, Cambridge, UK), anti-activator of RANKL (1:300, Abcam, Cambridge, UK), and anti-ostsoprotegrin (OPG) (1:300, Abcam, Cambridge, UK). Sections were subsequently incubated with Alexa Fluor 488-conjugated goat anti-rabbit IgG (1:400, Invitrogen, CA, USA), Alexa Fluor 568-conjugated goat anti-rabbit IgG (1:400, Invitrogen), Alexa Fluor 647-conjugated goat anti-rabbit IgG 652/668 nm (#AB2722623), Alexa Fluor 488-conjugated goat anti-rabbit IgG 578/603 nm (#AB2534072), Alexa Fluor 647-conjugated goat anti-mouse IgG 652/668 nm (#AB2687948, Abcam).

Images were captured with an upright fluorescence microscope (Zeiss Axio Imager A2, Zeiss, Germany) under the setup with appropriate excitation/emission wavelengths of the filters. A digital camera linked to a computer running Axioscope version 4 (Zeiss. RRID: SCR_002677) captured images. The total number of osteoclasts, RANKL-containing osteoblasts and osteocytes, and OPG-containing osteoblasts and osteocytes were counted at X200. The average fluorescence intensity expressed per square micrometer from each animal was counted using Image J software.

### Statistics

Inspectors blinded to the experimental conditions assessed the histopathological changes in each experiment. Statistical analyses were performed using GraphPad Prism 8 (GraphPad Software Inc., CA, USA). Data are expressed as the mean ± SD. Data were assessed for normality using the Shapiro‒Wilk test. For the gingival index, LPS and LTA concentrations, micro CT, and histological staining analysis, two-way ANOVA was conducted to assess group and time-after-surgery effects and interactions within each time block. Any significant main effects for group, time, or interaction were followed by Tukey's multiple-comparisons post hoc testing. *P*-values < 0.05 were considered statistically significant. For the overview comparison reported in **Table 3** (Day 28 only), we also provide effect sizes and false-discovery rate (FDR)-adjusted inferences to aid clinical interpretation. Between-group effect sizes were reported as Hedges' g (signed; treatment vs PD, small-sample corrected). Pairwise contrasts (EHBOT vs PD; LHBOT vs PD) used two-sided Welch's t-tests, with p values adjusted across all Table-3 comparisons by Benjamini-Hochberg to yield q values. This summary does not replace the factorial ANOVA but complements it by quantifying magnitude (g) and multiplicity-controlled evidence (q) for the experimentally salient contrasts at Day 28.

## Results

### HBOT prevents and reverses gingival injury and bacterial metabolite accumulation

The periodontitis (PD) and recovery (PD+RECOV) groups exhibited significantly higher gingival index (GI) values compared to the sham group. However, both early HBOT (PD+EHBOT) and late HBOT (PD+LHBOT) groups showed significantly reduced GI index and bleeding on probing (BOP) values compared to the PD group (**Fig. 1B, C**). Similarly, the PD group had significantly elevated periodontal concentrations of bacterial metabolites, including lipoteichoic acid (LTA) and lipopolysaccharide (LPS), at both day 7 and day 28 post-ligature. These increases were significantly attenuated in the PD+LHBOT group (**Fig. 1D, E**).

### HBOT reduces inflammatory cytokines and chemokines in the gingival tissue

To evaluate the gingival inflammatory milieu, we profiled a targeted cytokine/chemokine panel at days 14 and 28 across all groups. Representative arrays (**Fig. 2A**) and quantitative analyses (**Fig. 2B-C**) showed that ligature-induced periodontitis (PD) elicited marked upregulation of multiple mediators at day 28 versus sham, including CINC-1 (CXCL1), CINC-2α/β (CXCL3), soluble ICAM-1 (sICAM-1), IL-1α, IL-1β, IL-1RA, LIX (CXCL5), L-selectin (CD62L), thymus chemokine (CCL25), and TIMP-1, consistent with sustained chronic gingival inflammation.

Early HBOT (PD+EHBOT) significantly suppressed the expression of most inflammatory mediators at day 28, including CINC-1 (p < 0.0001), CINC-2α/β (p < 0.0001), sICAM-1 (p < 0.0001), IL-1α (p < 0.0001), IL-1β (p = 0.0253), IL-1ra (p < 0.0001), LIX (p < 0.0001), thymus chemokine (p < 0.0001), and TIMP-1 (p < 0.0001), and notably reduced L-selectin expression at day 14 (p = 0.0086) (**Fig. 2B-C**), indicating attenuated leukocyte recruitment in the early inflammatory phase. However, by day 28, L-selectin levels increased across all groups, including PD+EHBOT and PD+LHBOT, and were higher than those observed in the PD+RECOV group (all p < 0.0001), suggesting potential late-phase immune cell reactivation or vascular remodeling.

Compared with the PD+RECOV group, both HBOT-treated groups demonstrated greater reductions in inflammatory cell infiltration markers (e.g., IL-1ra, all p < 0.0001) and chemokines (e.g., thymus chemokine, all p < 0.0001), indicating that natural recovery alone was insufficient to resolve inflammatory signaling. Early HBOT (PD+EHBOT) significantly reduced thymus chemokine levels more effectively than natural recovery (PD+RECOV) (p = 0.0062), suggesting a more robust attenuation of lymphocyte recruitment and inflammatory signaling. This superior suppression was not as pronounced in the late HBOT group (PD+LHBOT), indicating a potential benefit of earlier intervention in resolving chronic chemokine-mediated inflammation.

Additionally, TIMP-1, a regulator of extracellular-matrix turnover, was elevated in PD and was returned toward sham with both HBOT regimens, early (p < 0.0001) and late (p = 0.0096), consistent with attenuation of inflammatory drive and a reduced need for a compensatory antiproteolytic response (**Fig. 2C**).

Collectively, the data indicate that HBOT, especially when initiated early, attenuates IL-1-driven osteoclastogenic signalling and the leukocyte-recruitment axis, encompassing neutrophil chemotaxis (CXCL1/CXCL3/CXCL5), lymphocyte-directed signalling (thymus chemokine or CCL25), and endothelial capture-roll-adhere dynamics (sICAM-1/L-selectin), while normalising matrix-regulatory tone (TIMP-1). The biological roles and study findings for each analyte are summarised in **Table 2**.

### HBOT prevents and reverses periodontal bone loss

**Figure 3:**
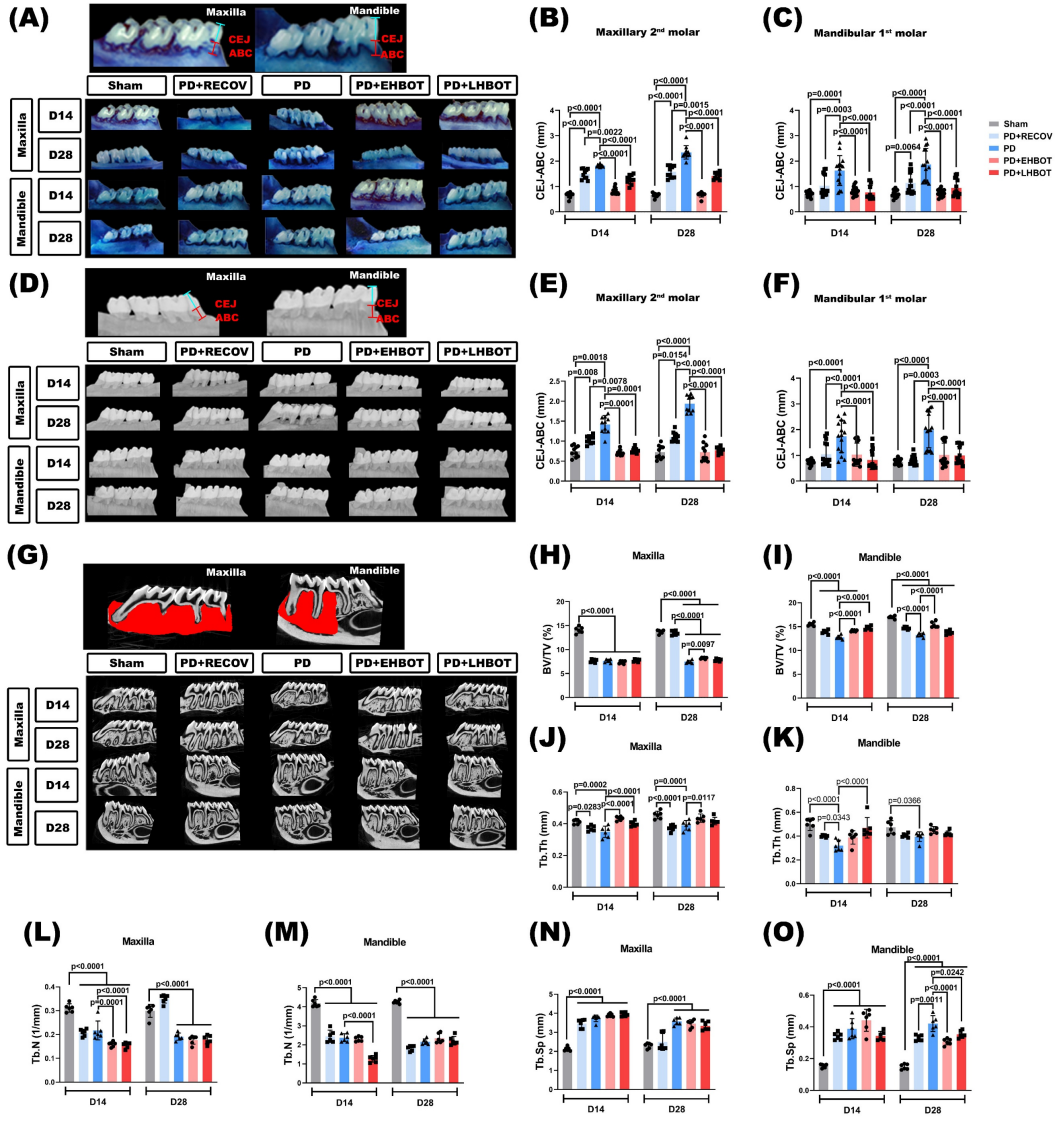
** Effect of hyperbaric oxygen therapy (HBOT) on alveolar bone loss and bone volumetric parameters in the maxillary second molar and mandibular first molar across study groups. (A)** Representative methylene blue-stained images showing alveolar bone loss. **(B, C)** Quantification of cementoenamel junction to alveolar bone crest (CEJ-ABC) distances in the maxillary second molar **(B)** and mandibular first molar **(C)**. **(D)** Representative micro-CT images illustrating alveolar bone loss. **(E, F)** CEJ-ABC distance measurements based on micro-CT analysis in the maxillary second molar **(E)** and mandibular first molar **(F)**. **(G)** Representative 3D micro-CT images of the maxillary second molar and mandibular first molar. Quantitative analysis of bone volumetric parameters, including bone volume/tissue volume (BV/TV), trabecular thickness (Tb.Th), trabecular separation (Tb.Sp), and trabecular number (Tb.N) in the maxilla **(H-K)** and mandible **(L-O)**, respectively. Abbreviations: PD, periodontitis; RECOV, recovery; EHBOT, early hyperbaric oxygen therapy; LHBOT, late hyperbaric oxygen therapy.

Periodontal bone loss was quantified using methylene blue staining (**Fig. 3A-C**) and micro-CT analysis (**Fig. 3D-O**). The length of the cementoenamel junction to the alveolar bone crest (CEJ-ABC) was measured at multiple predetermined sites (**Fig. 3A-F**). The PD and PD+RECOV groups, both maxillary and mandibular molar, exhibited significantly greater CEJ-ABC distances at all measured sites than the unligated controls (Sham), indicating substantial bone loss. In contrast, HBOT treatment in both the early (PD+EHBOT) and late (PD+LHBOT) intervention groups significantly reduced CEJ-ABC distances, suggesting effective mitigation of periodontal bone loss caused by ligature placement (**Fig. 3A-F**).

To evaluate alveolar bone loss and microarchitectural changes, high-resolution micro-CT was performed on maxillae and mandibles at days 14 and 28 (**Fig. 3G**). In the PD group, both maxillary and mandibular bone volume fraction (BV/TV) were significantly reduced compared to the sham group at both time points (**Fig. 3H-I**), reflecting severe periodontal bone loss. Trabecular thickness (Tb.Th) and trabecular number (Tb.N) were also significantly decreased (**Fig. 3J-M**), while trabecular separation (Tb.Sp) was markedly increased (**Fig. 3N-O**), indicating bone microstructural deterioration due to ligature-induced periodontitis.

Early HBOT (PD+EHBOT) significantly preserved bone volume and improved bone microarchitecture in both the maxilla and mandible. Specifically, PD+EHBOT restored BV/TV, Tb.Th, and Tb.N (**Fig. 3H-M**), and reduced Tb.Sp to levels closer to the sham group, particularly at day 28 (**Fig. 3N-O**). In contrast, the late HBOT group (PD+LHBOT) showed modest improvements, with significant but less pronounced recovery of BV/TV and Tb.Th, while Tb.N remained lower than the sham group.

Compared to the natural recovery group (PD+RECOV), both HBOT groups demonstrated superior preservation of bone volume and trabecular integrity. These data suggest that HBOT, particularly when initiated early, effectively prevents alveolar bone loss and structural degradation associated with periodontitis.

To aid comparison across endpoints, **Table 3** summarises Day-28 group means ± SD and signed Hedges' g versus PD (with BH-adjusted q values). Across maxillary and mandibular sites, early HBOT consistently produced larger absolute effect sizes than late HBOT for bone-preservation metrics (BV/TV, Tb.Th) and for inflammation-linked measures (CEJ-ABC, LTA/LPS, IL-1ra, thymus chemokine, TIMP-1), indicating a timing advantage.

### HBOT mitigates ICI and fibrosis in periodontal tissues

**Figure 4:**
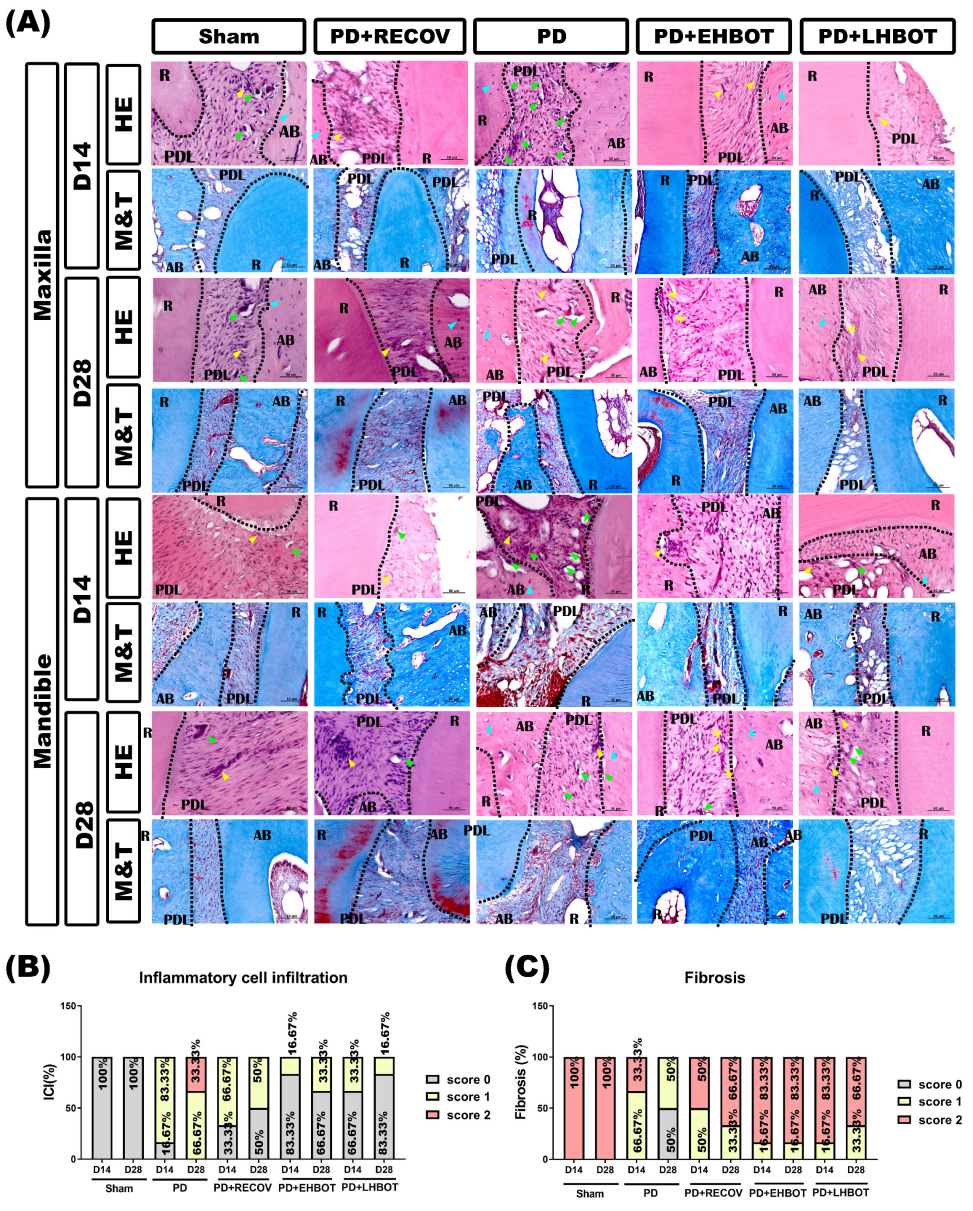
**Effect of HBOT on inflammatory cell infiltration (ICI) and fibrosis in the study groups. (A)** Representative images of the maxilla and mandible sections stained with hematoxylin-eosin (HE) and Masson's trichrome (M&T). Quantitative analysis of **(B)** inflammatory cell infiltration and **(C)** fibrosis ratio across the study groups.

Histological analysis using H&E and Masson's trichrome staining (**Fig. 4A**) showed higher inflammatory cell infiltration (ICI, **Fig. 4B**) scores in the PD group compared to the sham group. HBOT treatment significantly reduced ICI scores in both the maxilla and mandible, with the PD+EHBOT and PD+LHBOT groups showing a higher proportion of low-grade ICI (score 0). Fibrosis ratios, significantly reduced in the PD group compared to the sham group, were notably restored in the HBOT-treated groups (**Fig. 4C**).

### HBOT reduces the increased number of osteoclasts in periodontal tissues

**Figure 5:**
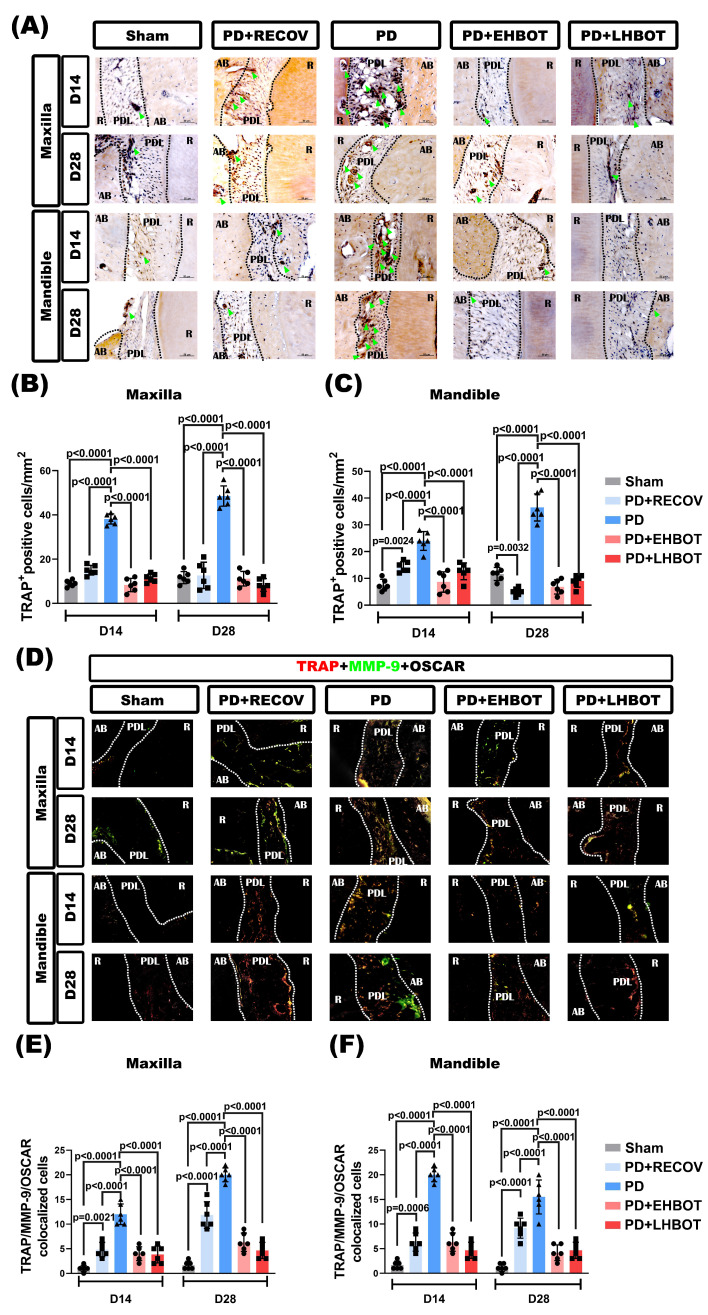
** Effect of HBOT on the number of TRAP^+^ osteoclasts in both the maxilla and mandible of the study groups. (A)** Representative immunohistochemical stained images of the study groups. **(B, C)** Each bar represents the TRAP+ cell in the maxilla and mandible. **(D)** Representative immunofluorescence TRAP^+^/MMP-9^+^/OSCAR^+^ osteoclast-stained images of the study groups. **(E, F)** Each bar represents the TRAP^+^/MMP-9^+^/OSCAR^+^ colocalized cells in the maxilla and mandible.

Immunohistochemical stainings revealed that compared to the sham group of rats, the PD or PD+RECOV group of rats had significantly higher numbers of TRAP-positive osteoclasts in the periodontal tissues of both maxilla and mandible (**Fig. 5A-C**). Again, immunofluorescence staining revealed that compared to the sham group of rats, the PD or PD+RECOV group of rats had significantly higher numbers of TRAP/MMP-9/OSCAR-colocalized osteoclasts in the periodontal tissues of both maxilla and mandible (**Fig. 5D-F**). However, compared to the PD group of rats, the PD+EHBOT or PD+LHBOT group of rats had significantly lower numbers of TRAP-positive osteoclasts (**Fig. 5A-C**) and TRAP/MMP-9/OSCAR-coloclaized osteoclasts in the periodontal tissues of both maxilla and mandible (**Fig. 5D-F**).

### HBOT reduces osteoclastogenic signaling and favors OPG-associated profiles in bone-lining cells

**Figure 6:**
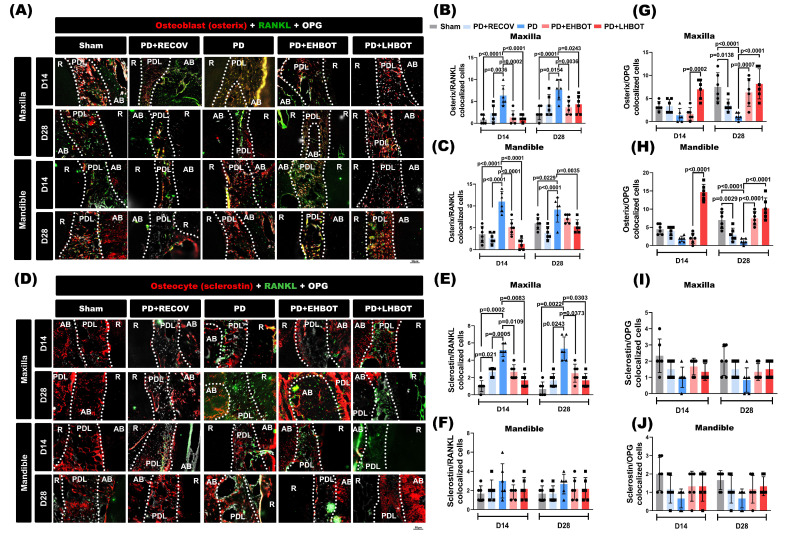
** HBOT restores the RANKL-OPG balance at bone-lining cells. (A)** Maxilla and** (D)** mandible: tri-channel composites from the same section and field of view (Osterix or Sclerostin in red; RANKL in green; OPG in gray). Dotted lines delineate root (R), periodontal ligament (PDL), and alveolar bone (AB).** (B-C)** Pairwise colocalization of Osterix+RANKL at D14 and D28. **(E-F)** Sclerostin+RANKL colocalization.** (G-H)** Osterix+OPG colocalization.** (I-J)** Sclerostin+OPG colocalization. Bars show mean ± SD; exact p values are indicated (two-way ANOVA with post-hoc testing; Day-28 overview in Table 3). Single-channel raw images (Osterix/Sclerostin, RANKL, OPG) and dual-channel overlays corresponding to the quantified pairs, together with acquisition metadata, are provided in** Supplementary Figures S2-S9.**

Representative tri-channel images acquired from the same section and field of view (Osterix/Sclerostin in red; RANKL in green; OPG in gray) are shown in maxilla and mandible at D14 and D28 (**Fig. 6A, 6D**). Quantitatively, ligature-induced periodontitis increased Osterix+RANKL and Sclerostin+RANKL colocalized cells in both the maxilla and mandible versus Sham, including after natural recovery (PD+RECOV) (**Fig. 6B-F**). HBOT, particularly when initiated early, significantly reduced these RANKL-associated readouts toward Sham levels (exact p values annotated in panels). Conversely, Osterix+OPG colocalization was decreased in PD/PD+RECOV and restored by HBOT, with a greater effect for early HBOT (**Fig. 6G-H**). Sclerostin+OPG did not show consistent between-group differences (**Fig. 6I-J**). Single- and dual-channel images supporting these analyses are provided in **Supplementary Figures S2-S9**.

## Discussion

Periodontitis is a prevalent inflammatory disease that causes the progressive destruction of tooth-supporting structures and leads to irreversible tooth loss. It is initiated by the accumulation of dental plaque, which fosters the growth of anaerobic bacteria that release pathogen-associated molecular patterns (PAMPs), such as LTA and LPS. These bacterial metabolites activate toll-like receptors (TLRs), particularly TLR2 and TLR4 39, triggering downstream signalling (e.g., NF-κB, MAPK) that promotes the release of pro-inflammatory cytokines and matrix-degrading enzymes, leading to bone resorption via osteoclast activation 1, 2, 28.

Our findings demonstrate that HBOT significantly mitigates periodontal bone loss in a rat model of ligature-induced periodontitis through multiple converging mechanisms. At the inflammatory level, HBOT reduced gingival levels of LTA and LPS and downregulated key cytokines and chemokines, including CXCL1 (CINC-1), CXCL3 (CINC-2α/β), CXCL5 (LIX), IL-1α, IL-1β, IL-1RA, sICAM-1, L-selectin, thymus chemokine (CCL25), and TIMP-1.

Interleukin-1 family cytokines play pivotal roles in the pathogenesis of periodontitis by regulating both inflammatory and bone-remodeling pathways. IL-1α is an early pro-inflammatory cytokine abundantly expressed in epithelial and resident immune cells. It promotes extracellular matrix degradation and enhances RANKL expression, thereby initiating osteoclastogenesis 40. IL-1β, a closely related cytokine, exerts potent effects on bone resorption by stimulating RANKL expression on osteoblasts and periodontal ligament cells 41, 42. Although IL-1β can also induce OPG, studies indicate that it predominantly enhances RANKL expression while either suppressing or insufficiently promoting OPG, leading to an elevated RANKL/OPG ratio and increased osteoclast activity 41-43. In our ligature-induced model, elevated IL-1β was associated with excessive osteoclastogenesis and alveolar bone loss. HBOT markedly reduced IL-1β levels and restored the RANKL/OPG balance, suggesting that suppression of IL-1 signalling may be a key mechanism underlying its bone-protective effects.

IL-1 receptor antagonist (IL-1RA) is an endogenous inhibitor that competes with IL-1α and IL-1β for receptor binding without activating downstream signalling; its elevation in periodontitis often reflects an inadequate compensatory response 41. The concurrent reduction of IL-1α, IL-1β, and IL-1RA following HBOT implies that this therapy promotes resolution rather than transient suppression, contributing to improved periodontal homeostasis.

TIMP-1, classically an inhibitor of matrix metalloproteinases and extracellular matrix degradation, was also elevated in the periodontitis group, likely a compensatory response to increased proteolytic activity. Beyond matrix regulation, TIMP-1 has been implicated in osteoblast differentiation and indirect modulation of RANKL/OPG, linking it to pathological bone remodelling 44, 45.

In addition to classical pro-inflammatory cytokines, our cytokine array analysis revealed significant upregulation of several chemokines in ligature-induced periodontitis, including thymus chemokine (CCL25), CINC-1 (CXCL1), and CINC-2α/β (CXCL3). Thymus chemokine, typically expressed in the small intestine and thymus, is involved in the recruitment of CCR9⁺ T cells and plays a role in mucosal immunity 46, 47. Its ectopic expression in inflamed gingival tissues suggests a potential role in sustaining chronic T cell-mediated inflammation within the periodontium. CINC-1 and CINC-2α/β, the rat homologs of human CXCL1 and CXCL3, are potent neutrophil chemoattractants that amplify the innate immune response by promoting leukocyte infiltration 48-51. While this response is initially protective, chronic overexpression contributes to excessive neutrophil-mediated tissue degradation through the release of proteolytic enzymes and reactive oxygen species, ultimately accelerating periodontal destruction 52, 53. Notably, HBOT significantly downregulated the expression of these chemokines, particularly CINC-1 and CINC-2α/β, and reduced thymus chemokine levels more effectively than natural recovery. These findings suggest that HBOT not only suppresses pro-inflammatory cytokines but also attenuates chemokine-driven leukocyte recruitment, thereby limiting immune cell infiltration and preserving periodontal tissue integrity.

Importantly, HBOT also significantly reduced the expression of LIX/CXCL5 and adhesion molecules such as sICAM-1 and L-selectin, which play pivotal roles in neutrophil recruitment, endothelial activation, and transmigration during gingival inflammation 52, 54-56. The suppression of LIX/CXCL5 by HBOT indicates attenuation of the chemotactic gradient necessary for leukocyte trafficking, thereby limiting excessive immune cell infiltration and associated tissue damage. Similarly, downregulation of sICAM-1 and L-selectin suggests decreased endothelial-leukocyte interactions, thereby reducing extravasation and amplifying the anti-inflammatory and tissue-protective effects of HBOT 48, 50, 52-55.

At the bone level, ligature-induced periodontitis elevated the RANKL/OPG ratio in osteoblasts and osteocytes, promoting osteoclastogenesis and bone resorption 6, 57. HBOT restored this balance by decreasing RANKL expression and increasing OPG levels, thereby reducing osteoclast numbers and preserving alveolar bone microarchitecture. These findings align with* in vitro* data showing that HBOT inhibits RANKL-induced osteoclast formation in RAW264.7 cells and PBMCs, with greater efficacy than hyperoxia or pressure alone 58. Our findings extend this understanding by demonstrating that HBOT directly modulates gingival inflammation, bacterial load, and bone remodelling* in vivo*.

Mechanistically, elevated tissue oxygen under HBOT can transiently rebalance redox signalling to favour cytoprotective programs while restraining pro-inflammatory transcription. The hyperoxic milieu elicits a controlled redox response that enhances Nrf2-dependent cytoprotective pathways while attenuating NF-κB/AP-1 activity, consistent with our observed reductions in IL-1α/β, CXCL5, ICAM-1, and L-selectin 59, 60. Hyperoxia also favors a shift from M1-like to pro-resolution M2-like phenotypes, limiting neutrophil chemoattractants and matrix-degrading signals 61. Concomitantly, decreased LTA/LPS levels suggest dampened TLR2/TLR4 engagement, lowering innate immune activation and leukocyte infiltration 62-64. At the bone interface, HBOT favors osteoblast/OPG-dominant signaling and constrains RANKL-driven osteoclastogenesis, thereby restoring the RANKL/OPG axis and limiting trabecular degradation 65, 66. These convergent effects provide a biologically coherent explanation for the multi-targeted anti-inflammatory and bone-protective benefits of HBOT in ligature-induced periodontitis.

### Clinical implications and practical positioning

From a translational standpoint, our data support HBOT as a time-sensitive adjunct to conventional care. Early initiation, operationalised here as Day 3 after ligature, was associated with larger anti-inflammatory and bone-preserving effects than later initiation. A pragmatic clinical hypothesis is to cluster HBOT sessions around initial SRP (e.g., 2.0 ATA, 60 min, 5-10 sessions over 2-3 weeks) to maximise microbial- and vascular-milieu benefits while inflammation is most labile, followed by short 'top-up' blocks for non-responders. Candidate bedside markers such as LTA/LPS and the RANKL/OPG ratio may help tailor dose and timing in future studies.

### Limitations and future directions

This is an animal model, and inter-species differences in pocket anatomy, microbiota, and host immunity limit direct extrapolation. Dose-response, durability, and safety should be tested in well-controlled clinical trials (e.g., SRP ± HBOT with biomarker-guided schedules), including high-risk strata (smokers, diabetes) to determine who benefits most.

Beyond suppressing osteoclast activity, HBOT has been shown to enhance fibroblast proliferation, collagen deposition, angiogenesis, and osteoblast function, all of which contribute to the healing of periodontal tissues 67, 68. Our findings extend this understanding by demonstrating that HBOT directly modulates gingival inflammation, bacterial load, and bone remodelling in vivo. Clinically, HBOT is a safe and non-invasive intervention already in use for treating osteomyelitis and osteonecrosis 69, 70. Its potential as an adjunctive therapy for refractory periodontitis is supported by its multi-target effects, attenuating microbial burden, dampening inflammation, reducing immune cell recruitment, and rebalancing osteoclast regulatory pathways. Future studies should optimise treatment parameters, explore cost-effectiveness, and assess efficacy in systemic comorbidities such as diabetes or tobacco use.

**Figure 7:**
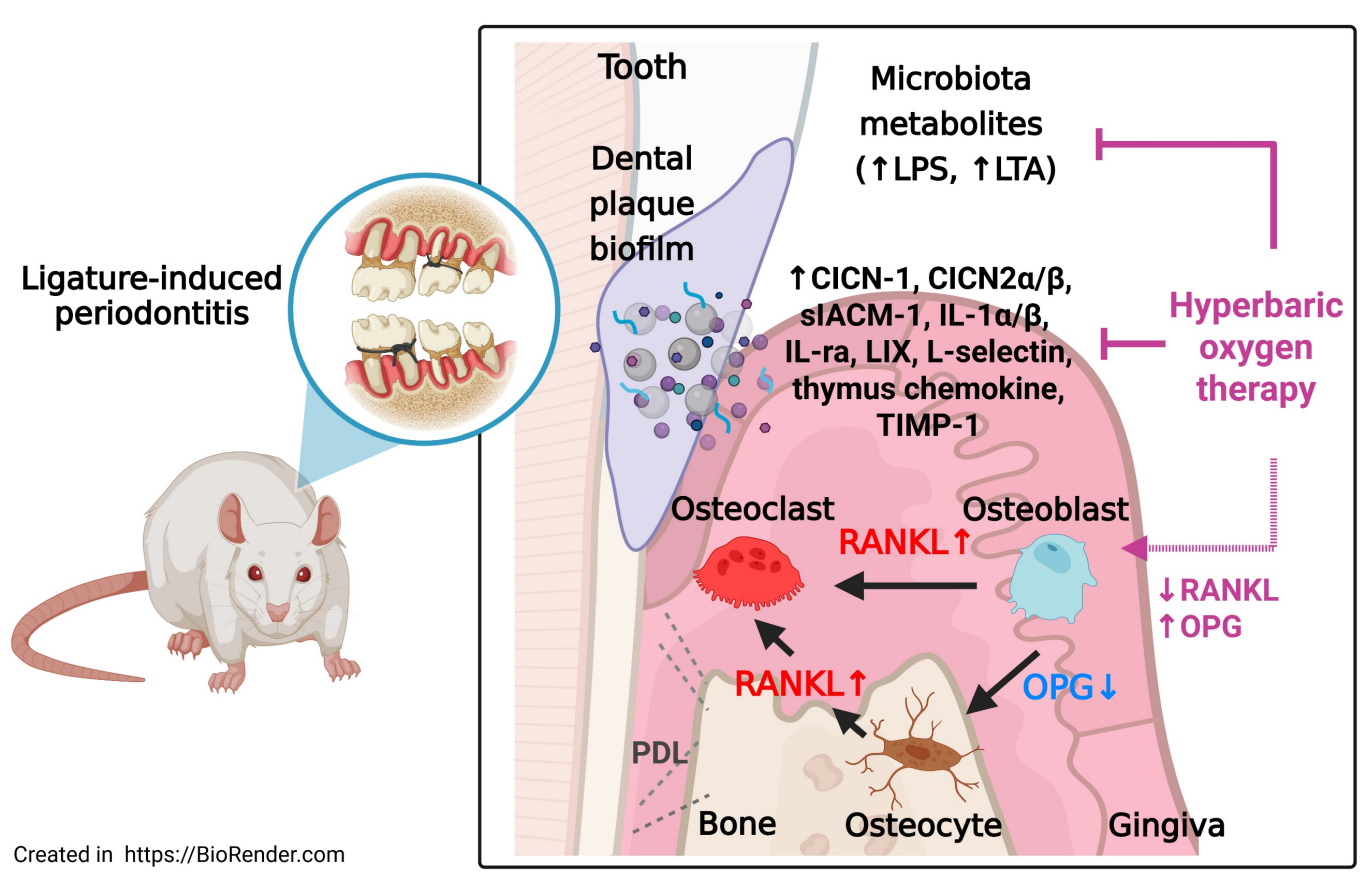
** Schematic summary of the effects of HBOT on ligature-induced periodontitis.** Ligature placement facilitates the formation of dental plaque biofilms, resulting in the release of microbiota metabolites, including lipopolysaccharides (LPS) and lipoteichoic acid (LTA). Ligature placements increased the number of RANKL-containing osteoblasts and RANKL-containing osteocytes but decreased the number of OPG-containing osteocytes and OPG-containing osteoblasts in the periodontal tissues of both the maxillary second molar and mandible first molar. The increased ratio between the RANKL and OPG expression in osteoblasts and osteocytes enhances osteoclast formation and results in gingival injury as well as periodontal bone loss. HBOT counteracts the formation of dental plaque biofilms, the release of microbial metabolites, the increased ratio between RANKL and OPG expression in osteoblasts and osteocytes, the formation of osteoclasts, and gingival injury, as well as periodontal bone loss caused by ligature placements. The red arrows indicate pathways driving osteoclastogenesis and tissue injury, while the blue arrows indicate the protective effects of HBOT on osteoclasts, osteocytes, and periodontal bone healing.

In summary (**Fig. 7**), HBOT mitigates periodontal destruction by: (1) reducing bacterial metabolites; (2) suppressing pro-inflammatory cytokines and chemokines; (3) downregulating adhesion molecules to limit immune infiltration; and (4) restoring the RANKL-OPG axis to inhibit osteoclastogenesis. These multi-faceted effects position HBOT as a promising adjunctive therapy targeting inflammation and bone remodelling in periodontitis.

## Supplementary Material

Supplementary figures.

## Figures and Tables

**Table 1:** Experimental groups, HBOT schedule, and assessment time points. NBA, normobaric air. HBOT was delivered once daily at 2.0 ATA for 60 min using 100% O₂. Early HBOT began on Day 3 after ligature (D3-D28; 26 sessions); late HBOT began on Day 7 (D7-D28; 22 sessions). Animals were assessed at Day 14 and Day 28.

Group	Ligature	HBOT regimen	Start day	Session count	Exposure per session	Assessment time points
Sham	No	None (NBA control)	-	-	21% O₂ at 1.0 ATA	Day 14, Day 28
PD+RECOV	7 days, then removed	None	-	-	21% O₂ at 1.0 ATA	Day 14, Day 28
PD	28 days	None	-	-	21% O₂ at 1.0 ATA	Day 14, Day 28
PD+EHBOT	28 days	100% O₂ at 2.0 ATA, 60 min/day	D3	26	Compression/decompression per manufacturer	Day 14, Day 28
PD+LHBOT	28 days	100% O₂ at 2.0 ATA, 60 min/day	D7	22	Compression/decompression per manufacturer	Day 14, Day 28

a. Animals not receiving HBOT were placed in the chamber under NBA for an equivalent duration to control for handling and environmental exposure. b. Exclusion criteria during exposures included persistent respiratory distress, signs of barotrauma, neurologic abnormalities, or features suggestive of oxygen toxicity; excluded animals were replaced to maintain prespecified group sizes.

**Table 2:** Cytokine/chemokine/adhesion targets profiled and their primary biological roles in periodontitis

Molecule (alias)	Canonical biological role in PD	Typical change in PD	Our study findings	Response to HBOT (this study)	Key refs
IL-1α	Early epithelial/immune-cell cytokine; ↑inflammatory cascade; drives ECM degradation, ↑RANKL	↑	↑	↓	40, 42, 71
IL-1β	Potent pro-resorptive cytokine; ↑CX3CL1; ↑RANKL and ↑MMPs on osteoblast/PDL cells; ↑PGE2 in fibroblast cells;↑RANKL/OPG ratio; stimulates osteoclastogenesis, leads to bone resorption	↑	↑	↓	42, 43, 71
IL-1RA	Endogenous antagonist of IL-1 signalling	Compensatory ↑ / variable	↑	↓ in parallel with IL-1α/β	40, 41
CINC-1 (CXCL1)	Neutrophil chemoattractant; amplifies innate influx	↑	↑	↓	48, 50, 72, 73
CINC-2α/β (CXCL3)	Neutrophil chemoattractant; overlaps CXCL1 functions	↑	↑	↓	48-50, 74
LIX (CXCL5)	LPS-inducible chemokine; recruits/activates neutrophils, monocytes, macrophages, and T lymphocytes	↑	↑	↓	52, 53, 72
thymus chemokine (CCL25)	CCR9+ T-cell, macrophages, and dendritic cells recruitment; mucosal immunity	↑	↑	↓	47, 75
sICAM-1	Endothelial/epithelial activation and intensified leukocyte recruitment at diseased sites; potent immunomodulation and anti-inflammation; levels correlate with clinical severity and fall after therapy	no change/ compensatory ↑	↑	↓	54, 76
L-selectin	Leukocyte rolling/trafficking; shedding modulates influx	↑	↑	↓	55, 77
TIMP-1	Inhibits MMPs; matrix preservation; also modulates osteoblast differentiation & RANKL/OPG indirectly	Often dysregulated/ compensatory ↑	↑	↓ / normalization	44, 45

**Table 3:** Overview of key endpoints at Day 28 (means ± SD) and effect sizes vs PD

Endpoint (Day)	Sham (mean±SD)	PD (mean±SD)	PD+RECOV (mean±SD)	PD+EHBOT (mean±SD)	PD+LHBOT (mean±SD)	Hedges' g (EHBOT vs PD)	*q* (EHBOT vs PD)	Hedges' g (LHBOT vs PD)	*q* (LHBOT vs PD)
Gingival index D28	0.00±0.00	1.63±0.52	0.38±0.52	0.25±0.46	0.38±0.52	-2.59	0.0025	-2.22	0.0004
CEJ-ABC (mm) MB-MAX D28	0.66±0.08	2.34±0.26	1.58±0.23	0.68±0.10	1.41±0.13	-7.78	<0.0001	-4.8	<0.0001
CEJ-ABC (mm) MB-Mand D28	0.71±0.14	1.14±0.42	1.88±0.63	0.76±0.14	0.95±0.33	-1.12	<0.0001	-0.46	<0.0001
CEJ-ABC (mm) μCT-MAX D28	0.72±0.17	1.94±0.21	1.10±0.12	0.72±0.23	0.77±0.08	-5.11	<0.0001	-6.80	<0.0001
CEJ-ABC (mm) μCT-Mand D28	0.75±0.11	1.99±0.69	0.82±0.17	1.02±0.46	0.99±0.34	-1.53	<0.0001	-1.70	<0.0001
BV/TV (%) Max-D28	13.69±0.39	7.46±0.32	13.45±0.45	8.20±0.16	7.82±0.27	+2.70	0.0005	+1.12	0.0352
BV/TV (%) Mand-D28	16.97±0.29	13.25±0.39	14.68±0.26	15.41±0.62	13.82±0.43	+3.85	<0.0001	+1.28	0.0158
Tb.Th (µm) Max-D28	0.45±0.02	0.39±0.03	0.37±0.02	0.44±0.03	0.42±0.02	+1.54	0.0022	+1.09	0.0364
Tb.Th (µm) Mand-D28	0.48±0.06	0.40±0.04	0.41±0.02	0.45±0.03	0.42±0.02	+1.31	0.1843	+0.58	0.5502
LTA (pg/ml) D28	160.41±87.92	697.66±122.06	410.94±43.6	172.3±91.76	284.27±43.59	-4.49	0.0021	-4.16	0.0021
LPS (pg/ml) D28	91.11±46.96	18646.76±3464.72	5236.34±807.73	914.64±388.81	3897.85±1007.97	-6.64	<0.0001	-5.34	0.0002
IL-1ra (pg/mg) D28	1.00±0.00	1.63±0.04	1.34±0.06	1.06±0.07	1.14±0.13	-9.23	<0.0001	-4.70	<0.0001
Thymus chemokine (AU) D28	1.04±0.06	1.67±0.06	1.33±0.07	1.04±0.06	1.26±0.14	-9.69	<0.0001	-3.51	<0.0001
TIMP-1(AU) D28	1.00±0.00	1.47±0.37	1.00±0.00	1.00±0.00	1.19±0.29	-1.66	<0.0001	-0.78	0.0009

Data are mean ± SD; n = 6 per group at Day 28. Groups: Sham, PD (periodontitis), RECOV (ligature removal + natural recovery), EHBOT (early HBOT; D3-D28), LHBOT (late HBOT; D7-D28). g = Hedges' g (signed effect size; treatment vs PD), computed from Cohen's d with small-sample correction; g > 0 indicates higher than PD (e.g., bone-preservation metrics), g < 0 indicates lower than PD (e.g., inflammatory/bone-loss markers). q = FDR-adjusted p (Benjamini-Hochberg) across all Table 3 pairwise contrasts. Abbreviations: CEJ-ABC = cementoenamel junction-alveolar bone crest distance; MB = methylene blue; μCT = micro-computed tomography; Max = maxilla; Mand = mandible; BV/TV = bone volume/total volume; Tb.Th = trabecular thickness; LTA = lipoteichoic acid; LPS = lipopolysaccharide; IL-1ra = interleukin-1 receptor antagonist; CCL25 = thymus chemokine; TIMP-1 = tissue inhibitor of metalloproteinases-1; AU = arbitrary units.
