# Supplementary material for: Hyperbaric oxygen protects against periodontal bone loss by modulating inflammation and bone remodeling via RANKL/OPG expression in ligature-induced periodontitis
Source: Int J Med Sci. 2026 Jan 1;23(2):478–96. doi: 10.7150/ijms.122857 (PMC12825118; doi:10.7150/ijms.122857)
Supplement: Supplementary file 1 — Supplementary figures. [file ijmsv23p0478s1.pdf]

Supplementary data

Hyperbaric oxygen protects against periodontal bone loss by modulating inflammation and bone remodeling via RANKL/OPG expression in ligature-induced periodontitis

Kang-Wei Tu, DDS

Department of Periodontics, Chi Mei Medical Center, Yongkang, Tainan 710, Taiwan

Department of Environmental and Occupational Health, College of Medicine, National Cheng Kung University, Tainan 70428, Taiwan.

Email: kangwei.periotu@gmail.com

Chien-Cheng Huang, MD, PhD

Department of Emergency Medicine, Chi Mei Medical Center, Tainan 710, Taiwan

School of Medicine, College of Medicine, National Sun Yat-sen University, Kaohsiung, Taiwan

Department of Emergency Medicine, Kaohsiung Medical University, Kaohsiung, Taiwan

Email: chienchenghuang@yahoo.com.tw

Mao-Tsun Lin, MD, PhD

Department of Medical Research, Chi Mei Medical Center, Tainan 710, Taiwan

891201@mail.chimei.org.tw

Ko-Chi Niu, MD, PhD

Department of Hyperbaric Oxygen, Chi Mei Medical Center, Tainan 710, Taiwan.

Email: kcniukc@gmail.com

Cheng-Hsien Lin, MD, PhD

Department of Medicine, Mackay Medical University, New Taipei City, Taiwan

Email: davidlin@mmu.edu.tw

Pi-Yu Chao, MS

Department of Medical Research, Chi Mei Medical Center, Tainan 710, Taiwan

piyuchao@gmail.com

Ching-Ping Chang, PhD

Department of Medical Research, Chi Mei Medical Center, Tainan 710, Taiwan

jessica.cpchang@gmail.com or a50831@mail.chimei.org.tw

ORCID iD: https://orcid.org/0000-0003-0890-9414

Jimmy Lian Ping Mau, DDS

Department of Periodontics, National Cheng Kung University Hospital, Tainan 704302, Taiwan.

Department of Periodontics, Tri-Service General Hospital, Taipei 114202, Taiwan.

Yuya Dental Clinic, Tainan 709020, Taiwan.

Department of Senior Services, Southern Taiwan University of Science and Technology, Tainan 710301, Taiwan.

lianpingmau@yahoo.com.tw

Corresponding author(s)

Ching-Ping Chang, PhD, Department of Medical Research, Chi Mei Medical Center, Tainan 710, Taiwan. Email: jessica.cpchang@gmail.com or a50831@mail.chimei.org.tw

Jimmy Lian Ping Mau, DDS, Department of Senior Services, Southern Taiwan University of Science and Technology, 710, Tainan, Taiwan. Email: lianpingmau@yahoo.com.tw

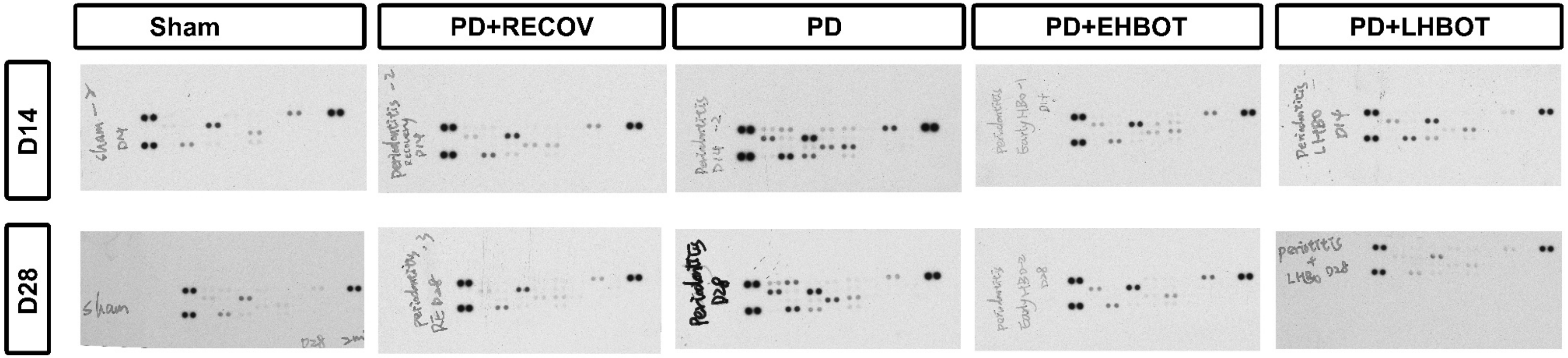

Figure S1. Original X-ray films for the cytokine dot array underlying Figure 2A (full membranes; single, intact exposures).

Rows indicate time points (D14, D28); columns indicate experimental groups (Sham, PD+RECOV, PD, PD+EHBOT, PD+LHBOT). Each panel shows one membrane from the Proteome Profiler Rat Cytokine Array Panel A (R&D Systems, ARY008), which is a fixed-coordinate dot array with duplicate capture spots per analyte and built-in reference/negative control spots at predefined positions. Films were acquired as single, intact exposures; the main-text Figure 2A was border-cropped only to remove blank margins (no content added, removed, or rearranged). The signal intensity of each dot reflects relative analyte abundance; the quantitative values plotted in Figure 2B-C were obtained by densitometry of the corresponding coordinates on these films.

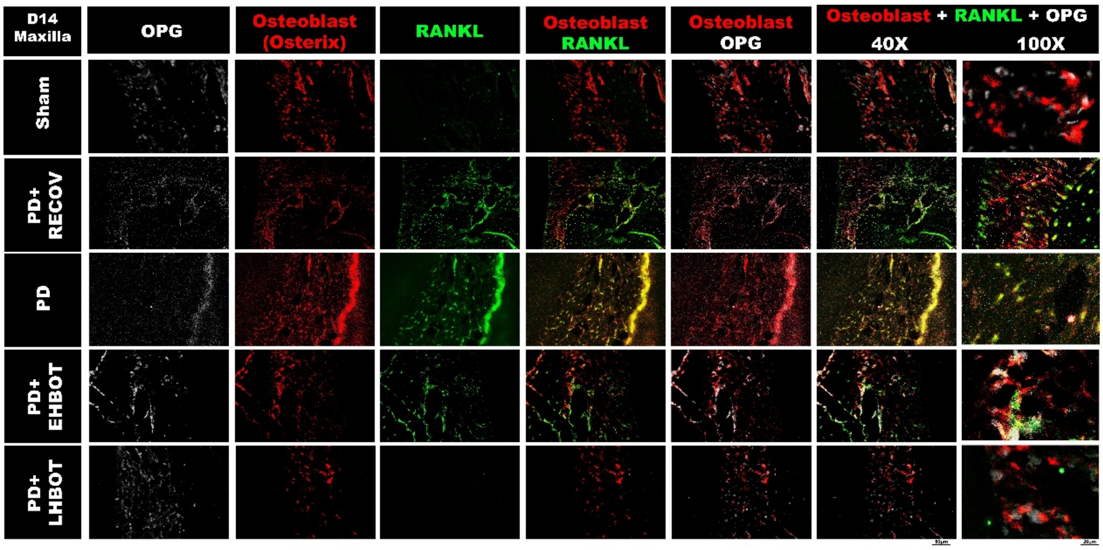

Figure S2. Day 14 — Maxilla | Osterix (osteoblast marker)/RANKL/OPG triple immunofluorescence (same section/field as Fig. 6A); single-, dual-, and tri-channel views supporting Osterix+RANKL and Osterix+OPG quantification (Fig. 6B-C, 6G-H).

Columns (left→right): single channel OPG (gray), single channel Osterix (red), single channel RANKL (green), dual-channel Osterix+RANKL, dual-channel Osterix+OPG, and tri-channel merge (Osterix red; RANKL green; OPG gray) at 40× and 100× (zoom-in). Rows: Sham, PD+RECOV, PD, PD+EHBOT, PD+LHBOT. Images were acquired from the same section and field of view with identical channel-specific settings; only global, linear adjustments were applied uniformly (no region-specific processing). The scale bars represent 50 µm (in the 40× images) and 25 µm (in the 100× images).

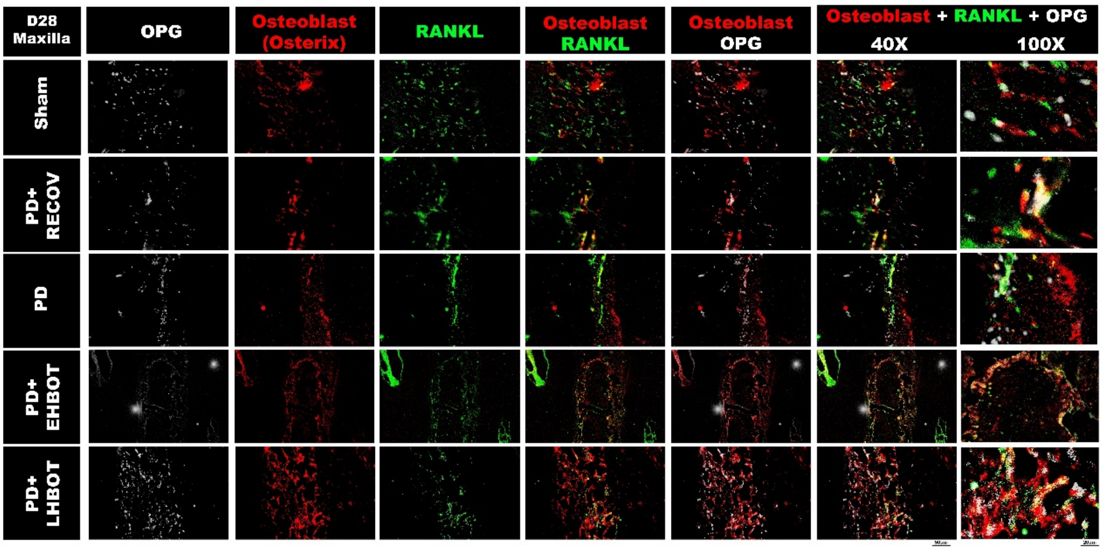

Figure S3. Day 28, Maxilla | Osterix (osteoblast marker)/RANKL/OPG triple immunofluorescence (same section/field as Fig. 6A); single-, dual-, and tri-channel views supporting Osterix+RANKL and Osterix+OPG quantification (Fig. 6B-C, 6G-H).

Columns (left→right): single channel OPG (gray), single channel Osterix (red), single channel RANKL (green), dual-channel Osterix+RANKL, dual-channel Osterix+OPG, and tri-channel merge (Osterix red; RANKL green; OPG gray) at 40× and 100× (zoom-in). Rows: Sham, PD+RECOV, PD, PD+EHBOT, PD+LHBOT. Images were acquired from the same section and field of view with identical channel-specific settings; only global, linear adjustments were applied uniformly (no region-specific processing). The scale bars represent 50 μm (in the 40× images) and 25 μm (in the 100× images).

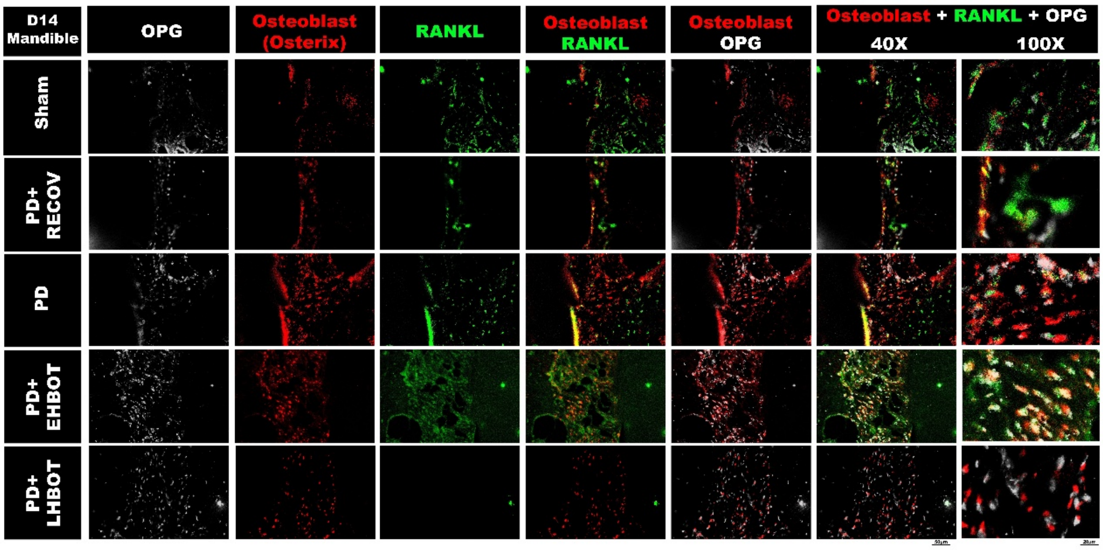

Figure S4. Day 14, Mandible | Osterix (osteoblast marker)/RANKL/OPG triple immunofluorescence (same section/field as Fig. 6A); single-, dual-, and tri-channel views supporting Osterix+RANKL and Osterix+OPG quantification (Fig. 6B-C, 6G-H).

Columns (left→right): single channel OPG (gray), single channel Osterix (red), single channel RANKL (green), dual-channel Osterix+RANKL, dual-channel Osterix+OPG, and tri-channel merge (Osterix red; RANKL green; OPG gray) at 40× and 100× (zoom-in). Rows: Sham, PD+RECOV, PD, PD+EHBOT, PD+LHBOT. Images were acquired from the same section and field of view with identical channel-specific settings; only global, linear adjustments were applied uniformly (no region-specific processing). The scale bars represent 50 μm (in the 40× images) and 25 μm (in the 100× images).

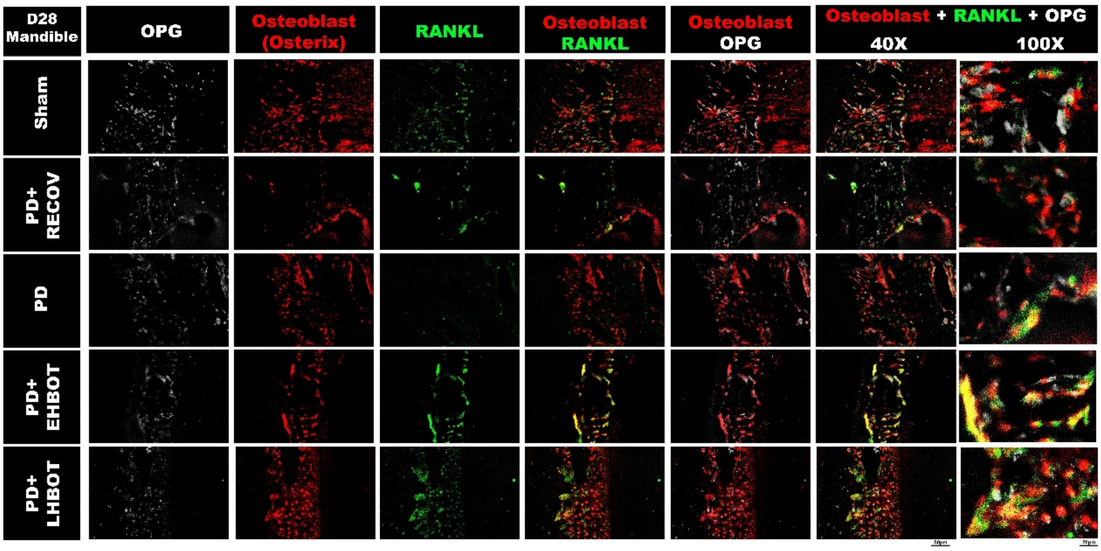

Figure S5. Day 28, Mandible | Osterix (osteoblast marker)/RANKL/OPG triple immunofluorescence (same section/field as Fig. 6A); single-, dual-, and tri-channel views supporting Osterix+RANKL and Osterix+OPG quantification (Fig. 6B-C, 6G-H).

Columns (left→right): single channel OPG (gray), single channel Osterix (red), single channel RANKL (green), dual-channel Osterix+RANKL, dual-channel Osterix+OPG, and tri-channel merge (Osterix red; RANKL green; OPG gray) at 40× and 100× (zoom-in). Rows: Sham, PD+RECOV, PD, PD+EHBOT, PD+LHBOT. Images were acquired from the same section and field of view with identical channel-specific settings; only global, linear adjustments were applied uniformly (no region-specific processing). The scale bars represent 50 µm (in the 40× images) and 25 µm (in the 100× images).

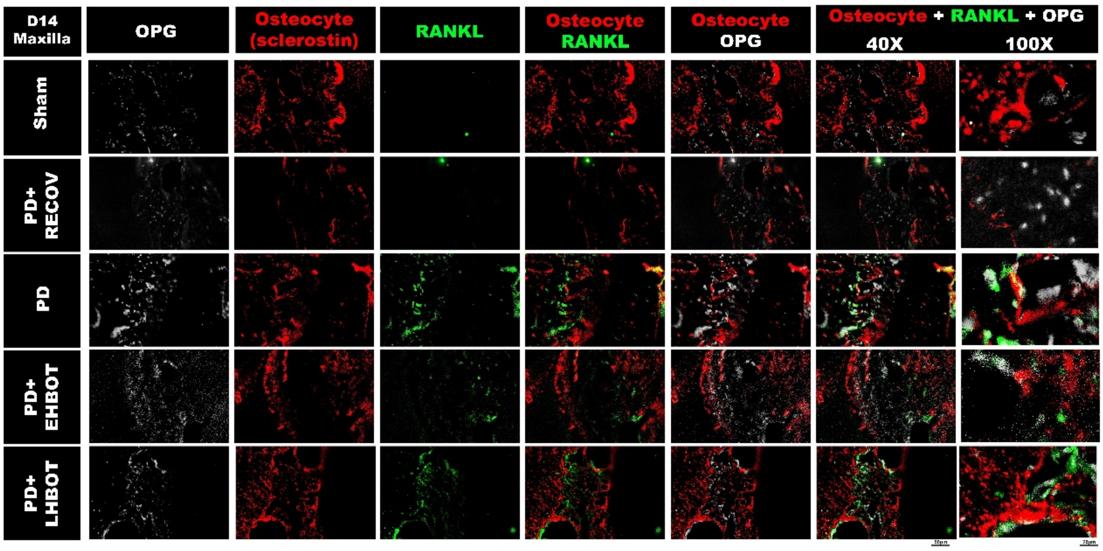

Figure S6. Day 14 — Maxilla | Sclerostin (osteocyte marker)/RANKL/OPG triple immunofluorescence (same section/field as Fig. 6D); single-, dual-, and tri-channel views supporting Sclerostin +RANKL and Sclerostin +OPG quantification (Fig. 6E-F, 6I-J).

Columns (left→right): single channel OPG (gray), single channel Sclerostin (red), single channel RANKL (green), dual-channel Sclerostin +RANKL, dual-channel Sclerostin +OPG, and tri-channel merge (Sclerostin red; RANKL green; OPG gray) at 40× and 100× (zoom-in). Rows: Sham, PD+RECOV, PD, PD+EHBOT, PD+LHBOT. Images were acquired from the same section and field of view with identical channel-specific settings; only global, linear adjustments were applied uniformly (no region-specific processing). The scale bars represent 50 µm (in the 40× images) and 25 µm (in the 100× images).

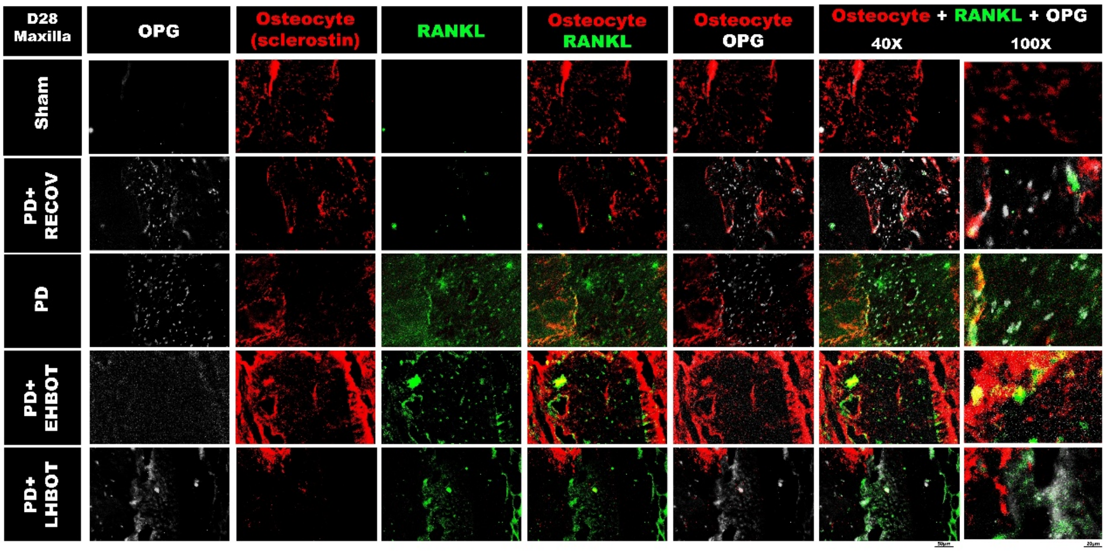

Figure S7. Day 28 — Maxilla | Sclerostin (osteocyte marker)/RANKL/OPG triple immunofluorescence (same section/field as Fig. 6D); single-, dual-, and tri-channel views supporting Sclerostin +RANKL and Sclerostin +OPG quantification (Fig. 6E-F, 6I-J).

Columns (left→right): single channel OPG (gray), single channel Sclerostin (red), single channel RANKL (green), dual-channel Sclerostin +RANKL, dual-channel Sclerostin +OPG, and tri-channel merge (Sclerostin red; RANKL green; OPG gray) at 40× and 100× (zoom-in). Rows: Sham, PD+RECOV, PD, PD+EHBOT, PD+LHBOT. Images were acquired from the same section and field of view with identical channel-specific settings; only global, linear adjustments were applied uniformly (no region-specific processing). The scale bars represent 50 µm (in the 40× images) and 25 µm (in the 100× images).

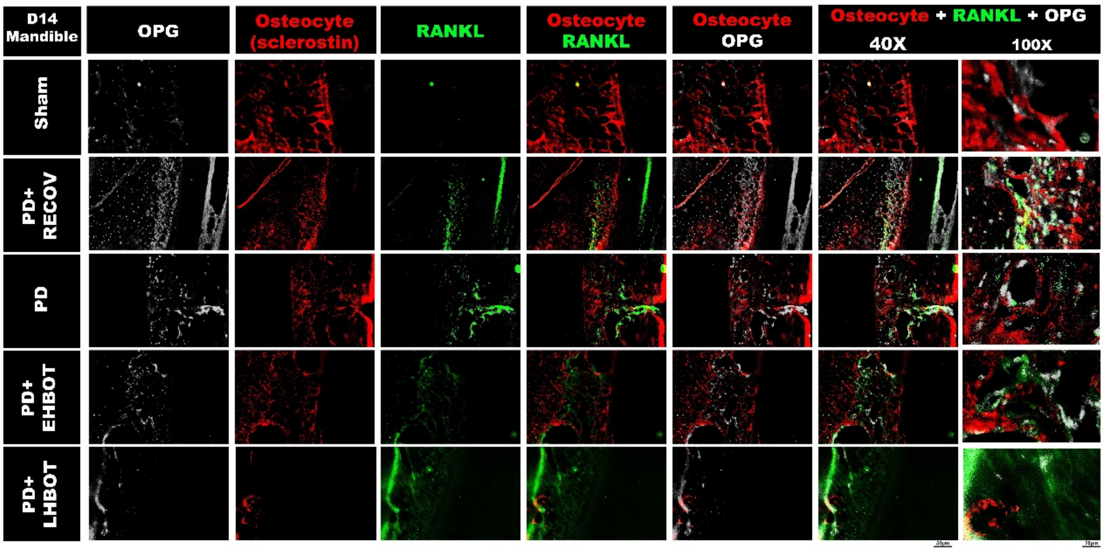

Figure S8. Day 14 — Mandible | Sclerostin (osteocyte marker)/RANKL/OPG triple immunofluorescence (same section/field as Fig. 6D); single-, dual-, and tri-channel views supporting Sclerostin +RANKL and Sclerostin +OPG quantification (Fig. 6E-F, 6I-J).

Columns (left→right): single channel OPG (gray), single channel Sclerostin (red), single channel RANKL (green), dual-channel Sclerostin +RANKL, dual-channel Sclerostin +OPG, and tri-channel merge (Sclerostin red; RANKL green; OPG gray) at 40× and 100× (zoom-in). Rows: Sham, PD+RECOV, PD, PD+EHBOT, PD+LHBOT. Images were acquired from the same section and field of view with identical channel-specific settings; only global, linear adjustments were applied uniformly (no region-specific processing). The scale bars represent 50 µm (in the 40× images) and 25 µm (in the 100× images).

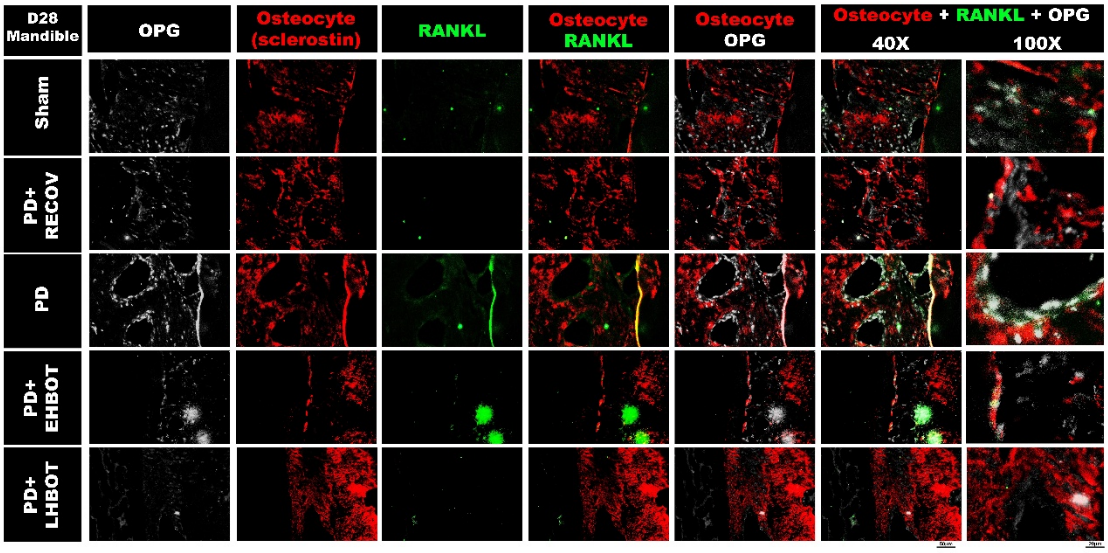

Figure S9. Day 28 — Mandible | Sclerostin (osteocyte marker)/RANKL/OPG triple immunofluorescence (same section/field as Fig. 6D); single-, dual-, and tri-channel views supporting Sclerostin +RANKL and Sclerostin +OPG quantification (Fig. 6E-F, 6I-J).

Columns (left→right): single channel OPG (gray), single channel Sclerostin (red), single channel RANKL (green), dual-channel Sclerostin +RANKL, dual-channel Sclerostin +OPG, and tri-channel merge (Sclerostin red; RANKL green; OPG gray) at 40× and 100× (zoom-in). Rows: Sham, PD+RECOV, PD, PD+EHBOT, PD+LHBOT. Images were acquired from the same section and field of view with identical channel-specific settings; only global, linear adjustments were applied uniformly (no region-specific processing). The scale bars represent 50 µm (in the 40× images) and 25 µm (in the 100× images).
